# Miscellany

**Published:** 1901-05

**Authors:** 


### MISCELLANY.

THE ST. PAUL MEETING AND YELLOWSTONE PARK.—Arrangements have been completed for an excursion of the members of the American Medical Association to Yellowstone Park. The committee of arrangements has finally succeeded in pursuading the officials to open up the park a week earlier than usual in order to accommodate the association. A special train will be run from St. Paul to the Yellowstone Park and the railroad officials have promised to do everything in their power to make it satisfactory to all concerned. The rates will be very low, but how low can not at this time be definitely stated.

---

RUDOLF VIRCHOW FUND.—The following has been sent to the American medical profession: On October 13, 1901, Rudolf Virchow will be 80 years old. When he completed his 70th year, a fund was started in his honor to enable the great master to facilitate scientific research by establishing scholarship, and by encouraging special medical and biological studies. Contributions to that "Rudolf Virchow Fund" were furnished by those in all countries interested in progressive medicine, as a homage to the man whose name is always certain to rouse admiration and enthusiasm.

In Berlin a large committee containing among others the names of A. Bastian, v. Coler, A. Entenburg, B. Fränkel, O. Israel, Fr. König, C. Posner and W. Waldeyer has been formed to call for contributions which are to be added to the original "Rudolf Virchow Fund" so as to increase its efficiency. The committee expresses the opinion that in no better way, and in none more agreeable to the great leader of modern medicine, can his eightieth birthday be celebrated, and ask for the sympathy and coöperation of all those engaged in the study and practice of scientific medicine all over the globe.

The undersigned have formed a sub-committee for the purpose of making the American profession acquainted with the intentions of the Berlin Committee, and urge their colleagues to participate in honoring the very man who has done more, these fifty years, than any other to make medicine a science and international.

Subscriptions should be sent to their secretary, who will receipt therefor.

CHARLES A. L. REED, President of the American Medical Association.
HENRY P. BOWDITCH. President of the Congress of American Physicians and Surgeons.
WILLIAM H. WELCH, Johns Hopkins University.
ROBERT F. WEIR, President of the New York Academy of Medicine.
A. JACOBI, 110 West 34th Street, New York, Secretary.

